# Hyperelastic and Stacked Ensemble-Driven Predictive Modeling of PEMFC Gaskets Under Thermal and Chemical Aging

**DOI:** 10.3390/ma17225675

**Published:** 2024-11-20

**Authors:** Su-Yeon Park, Akeem Bayo Kareem, Toyyeebah Ajibola Mustapha, Woo-Jeong Joo, Jang-Wook Hur

**Affiliations:** 1 Department of Aeronautics, Mechanical and Electronic Convergence Engineering, Kumoh National Institute of Technology, 61 Daehak-ro, Gumi-si 39177, Republic of Korea; 20246039@kumoh.ac.kr (S.-Y.P.); 20216004@kumoh.ac.kr (A.B.K.); 2 Department of Mathematics, College of Natural Science, Kyungpook National University, 80 Daehak-ro, Buk-gu, Daegu 41566, Republic of Korea; mustaphatoyyeebah@knu.ac.kr; 3 Pyung Hwa Oil Seal Co., Ltd., 597, Nongong-ro, Nongong-eup, Dalseong-gun, Daegu 42982, Republic of Korea; sorim2wn@ph.co.kr

**Keywords:** aging effects, deformation analysis, ensemble model, fuel cell reliability, gasket materials, hyperelastic models, PEMFC, predictive modeling, stress distribution

## Abstract

This study comprehensively investigates the stress distribution and aging effects in Ethylene Propylene Diene Monomer (EPDM) and Liquid Silicone Rubber (LSR) gasket materials through a novel integration of hyperelastic modeling and advanced machine learning techniques. By employing the Mooney–Rivlin, Ogden, and Yeoh hyperelastic models, we evaluated the mechanical behavior of EPDM and LSR under conditions of no aging, heat aging, and combined heat- and sulfuric-acid exposure. Each model revealed distinct sensitivities to stress distribution and material deformation, with peak von Mises stress values indicating that LSR experiences higher internal stress than EPDM across all conditions. For instance, without aging, LSR shows a von Mises stress of 24.17 MPa compared to 14.96 MPa for EPDM, while under heat and sulfuric acid exposure, LSR still exhibits higher stress values, showcasing its resilience under extreme conditions. Additionally, the ensemble learning approach achieved a classification accuracy of 98% for LSR and 84% for EPDM in predicting aging effects, underscoring the robustness of our predictive framework. These findings offer practical implications for selecting suitable gasket materials and developing predictive maintenance strategies in industrial applications, such as fuel cells, where material integrity under stress and aging is paramount.

## 1. Introduction

Sealing materials’ durability and mechanical performance are essential for the long-term efficiency of proton exchange membrane fuel cells (PEMFCs), particularly under extreme environmental conditions [1]. Ethylene Propylene Diene Monomer (EPDM) and Liquid Silicone Rubber (LSR) are widely used as gasket materials in PEMFCs because of their flexibility and resilience under mechanical loads [2]. However, these materials are prone to degradation from aging conditions, such as prolonged exposure to heat and sulfuric acid, which can compromise PEMFC performance and increase the risk of hydrogen fuel leaks—an important safety concern, particularly in hydrogen vehicles or fuel-cell electric vehicle (FCEV) applications [3,4].

Gaskets are critical for maintaining PEMFC integrity by preventing leaks and ensuring optimal operation under varying pressures, extreme temperatures, and mechanical stresses [5]. Given PEMFCs’ prominent role in hydrogen fuel-cell technology as a clean energy solution, especially in electric vehicles, their reliability heavily depends on the durability of these gasket materials [6]. Addressing the challenges posed by gasket degradation requires advanced modeling and testing techniques to predict performance, optimize material selection, and ultimately enhance PEMFC safety and efficiency. Significant progress has been made in advancing gasket materials, with EPDM and LSR showing promise due to their mechanical stability and resistance to chemical degradation [7,8]. This study specifically investigates the performance of gasket materials (EPDM and LSR) at 95 °C, representing high-temperature operational conditions typical in PEMFC systems, rather than low-temperature or cold-start conditions.

Thermal management is a crucial factor in PEMFC systems to maintain optimal operational temperatures, particularly for gasket materials that are susceptible to degradation under thermal stress. Effective thermal regulation minimizes overheating, which can accelerate material aging and reduce the durability of critical components. Recent studies have highlighted various approaches for managing thermal gradients, such as liquid cooling systems, nanofluid integration, and advanced flow field designs that enhance heat dissipation and maintain consistent temperatures across the fuel-cell stack [9,10,11,12]. These advancements improve the efficiency and longevity of PEMFCs and support the structural integrity of gasket materials, especially in automotive applications where both space and weight constraints are essential. Our research emphasizes the impact of sustained high temperatures, such as 95 °C, on the degradation of EPDM and LSR gaskets, as this aligns with the typical operational demands of PEMFC systems in high-temperature environments. Cold-start scenarios, which pertain to lower temperature thresholds, are beyond the scope of this study.

Recent studies underscore the importance of optimizing gasket design and material composition to enhance durability in PEMFCs. For instance, Yoo et al. demonstrated that half-circular gasket designs provide superior airtightness and durability even at −40 °C, making them suitable for PEMFCs operating in extreme conditions [13]. Sim et al. explored silica surface treatments to reduce silicon leaching, thereby minimizing contamination and preserving PEMFC membrane integrity for extended operation [14]. Complementary research by Fan et al. focused on system-level PEMFC design, integrating components like bipolar plates and gas diffusion layers to improve mass transfer and thermal management, which further enhances fuel-cell stack durability [15]. Furthermore, Wang et al. introduced a rate-dependent aging constitutive model for EPDM rubber, which effectively captures the effects of strain rate, aging time, and temperature, providing a framework for predicting long-term gasket performance in PEMFCs [16].

Moreover, Li et al. examined the aging behavior of EPDM, LSR, and fluorine rubber (FPM) under high- and low-temperature cycling, showing that temperature fluctuations can induce fatigue failure in rubber materials, increasing compression set—an outcome especially relevant for PEMFC gaskets under cyclic temperature conditions. Li et al.’s study introduced an accelerated aging test, offering a faster, cost-effective method to predict the service life of rubber seals under cyclic temperatures [17].

Hyperelastic models, such as Mooney–Rivlin, Yeoh, and Ogden, provide valuable insights into the non-linear elastic behavior of elastomers like EPDM and LSR. These models enable detailed stress–strain analysis under operational conditions. Although hyperelastic models have been individually researched, few studies comprehensively compare these models under different aging types and materials. This study investigates EPDM and LSR mechanical behavior across various aging conditions using these three models, focusing on von Mises stress, contact stress, and deformation at critical points in a PEMFC configuration. Through comparative analysis, this work develops predictive models to forecast material failure or degradation, which are essential for effective maintenance strategies and improved PEMFC performance and reliability.

- This study makes a unique contribution by comprehensively assessing EPDM and LSR material performance under simulated PEMFC operational stresses through a novel comparison of hyperelastic modeling methods under varied aging conditions.
- Unlike previous studies, this paper evaluates the models’ effectiveness in stress distribution and integrates predictive ensemble machine learning methods to classify aging effects.
- This approach advances predictive maintenance strategies, supporting improved material selection for PEMFC gaskets with enhanced durability and reliability.

These contributions advance PEMFC safety and durability by applying predictive modeling and data-driven maintenance strategies. They underline the potential of this research to inform more resilient gasket designs and efficient PEMFC maintenance practices. The remainder of this study is structured as follows: Section 2 presents the background study and related works on PEMFC gasket materials, hyperelastic modeling, and ensemble learning. Section 3 and Section 4 describe the methodology framework for modeling the mechanical performance and predictive maintenance of EPDM and LSR materials. Section 5 provides the study’s results, discussion, and limitations. Finally, Section 6 offers the concluding remarks and potential future directions.

## 2. Background Study and Literature Review

### 2.1. Hyperelastic Material Models

Hyperelastic models are commonly used to describe the non-linear stress–strain behavior of elastomers, which undergo significant deformations under load. Among the prominent models are Mooney–Rivlin, Yeoh, and Ogden, each offering unique advantages for modeling elastomers under different conditions.

#### 2.1.1. Mooney–Rivlin Model

The Mooney–Rivlin model assumes that the strain energy density *W* depends on the first two invariants of the deformation tensor, making it suitable for materials experiencing moderate strains. The strain energy function for the Mooney–Rivlin model is given by:

(1) $$W\;=\;C_{10}\left(I_{1}-3\right)+C_{01}\left(I_{2}-3\right)$$

where $C_{10}$ and $C_{01}$ are material constants, and $I_{1}$ and $I_{2}$ are the first and second invariants of the deformation tensor, defined as:

(2) $$I_{1}\;=\;\lambda_{1}^{2}+\lambda_{2}^{2}+\lambda_{3}^{2},\;I_{2}\;=\;\lambda_{1}^{2}\lambda_{2}^{2}+\lambda_{2}^{2}\lambda_{3}^{2}+\lambda_{3}^{2}\lambda_{1}^{2}$$

where $\lambda_{1}$, $\lambda_{2}$, and $\lambda_{3}$ are the principal stretches. Due to its simplicity, this model is widely used in finite element analysis (FEA) for sealing materials in PEMFCs. However, under extreme deformations or aging conditions (e.g., exposure to high temperature or acid), variations in $C_{10}$ and $C_{01}$ might reflect the material’s degradation, reducing the model’s accuracy [18,19].

#### 2.1.2. Yeoh Model

The Yeoh model extends the Mooney–Rivlin formulation by relying solely on the deformation tensor’s first invariant $I_{1}$, making it particularly effective for materials undergoing large strains. The strain energy function is expressed as:

(3) $$W\;=\;C_{10}\left(I_{1}-3\right)+C_{20}{\left(I_{1}-3\right)}^{2}+C_{30}{\left(I_{1}-3\right)}^{3}$$

where $C_{10}$, $C_{20}$, and $C_{30}$ are material constants. Including higher-order terms enables the Yeoh model to capture non-linear material responses more accurately. For applications involving high deformation, such as PEMFC gaskets made from EPDM and LSR, the flexibility of the Yeoh model in handling significant strain variations is advantageous. In the context of aging, the sensitivity of the $C_{20}$ and $C_{30}$ parameters may provide insights into the degree of degradation under conditions like heat and chemical exposure [20,21].

#### 2.1.3. Ogden Model

The Ogden model is based on principal stretches $\lambda_{i}$ rather than invariants, making it suitable for materials subjected to extreme loads and capable of modeling anisotropic behavior. The strain energy density function is defined as:

(4) $$W\;=\;\sum_{p=1}^{N}\frac{\mu_{p}}{\alpha_{p}}\left(\lambda_{1}^{\alpha_{p}}+\lambda_{2}^{\alpha_{p}}+\lambda_{3}^{\alpha_{p}}-3\right)$$

where $\mu_{p}$ and $\alpha_{p}$ are material parameters, and *N* represents the number of terms in the series. The choice of $\mu_{p}$ and $\alpha_{p}$ enables the Ogden model to represent complex material behaviors across various strain levels, but it requires significant computational resources. In our study, the Ogden model’s parameters are tuned to capture the effects of aging, with variations in $\alpha_{p}$ reflecting stiffness changes in materials exposed to harsh environments. This model is particularly effective for simulating the severe deformation of PEMFC gasket materials under combined thermal and chemical stresses [22,23].

### 2.2. Aging Effects on Elastomers

Elastomers like EPDM and LSR are favored in PEMFCs due to their flexibility and environmental resistance. However, aging, induced by thermal and chemical exposure, alters their mechanical properties, impacting their performance in sealing applications. Thermal and chemical aging primarily affect stiffness, tensile strength, and deformation characteristics, influencing stress distribution in these materials. Table 1 summarizes key studies on thermal and chemical aging effects on EPDM and LSR. High temperatures increase elastomer stiffness and reduce flexibility, which impacts contact, and von Mises stress in PEMFC gaskets, where mechanical integrity is vital. Mooney–Rivlin and Yeoh’s models have been widely applied to assess mechanical changes from thermal aging [24,25,26]. Acidic exposure, particularly sulfuric acid, degrades elastomers at the molecular level, reducing structural integrity. Studies indicate that combined thermal and chemical aging exacerbates degradation, impacting von Mises and contact stress distributions, especially in PEMFC gaskets [27,28]. In PEMFCs, elastomeric gaskets maintain structural integrity and prevent gas leakage, which is crucial for safety and efficiency [29]. EPDM and LSR gaskets are favored for their thermal and chemical degradation resistance. Hyperelastic models, such as Mooney–Rivlin, Yeoh, and Ogden, are employed to predict their mechanical response under varying loads and aging conditions. By simulating stress–strain relationships and deformation characteristics, these models enable accurate predictions of material performance over time, enhancing PEMFC reliability in demanding conditions [30]. Integrating hyperelastic models with predictive maintenance strategies offers a proactive approach to identifying gasket degradation. Analyzing aging effects through predictive models enables the early detection of material failure risks. Combined with a digital twin framework, these models provide real-time monitoring capabilities, allowing for timely maintenance interventions that increase PEMFC system longevity.

### 2.3. Ensemble Learning

EL improves predictive accuracy by combining multiple base models to reduce bias and variance and improve generalization. This study explores bagging, boosting, and stacking methods in EL, each contributing unique advantages to predictive modeling [31,32,33,34,35]:

- Bagging (Bootstrap Aggregating): generates multiple model versions by training on random data subsets, reducing variance. RF is a standard bagging algorithm known for stabilizing high-variance models.
- Boosting: builds models iteratively to correct errors from previous iterations, focusing on hard-to-predict instances. Techniques like AdaBoost and Gradient Boosting effectively address complex data patterns, though they may be prone to overfitting if not correctly regulated.
- Stacking: uses a meta-learner to combine predictions from multiple base learners, allowing models of different types (e.g., SVM, RF) to complement each other’s strengths, enhancing accuracy and robustness.

**Table 1:** Summary of recent studies on thermal and chemical aging of EPDM and LSR materials.

Study	Aging Type	Aging Condition	Performance Results
Thermal Aging of EPDM at 100 °C [36]	Thermal	130 °C, 145 °C, and 160 °C, with aging periods of up to 3072 h (130 °C), 768 h (145 °C), and 288 h (160 °C)	Breakdown strength decreased by 13.3% (130 °C), 21.2% (145 °C), and 22.5% (160 °C). Thermal degradation, chain breaking, and the generation of oxygen-containing groups led to reduced thermal stability. Initial decomposition temperature decreased by 11.35% after 288 h at 160 °C.
Acid and Thermal Aging of HTV Silicone Rubber [37]	Acid + Thermal	80 °C, Nitric Acid (pH = 1)	Significant cracking, reduced tensile strength from 4.58 MPa to 2.07 MPa, fracture strain reduced from 470% to 130%, thermal stability reduced by 30 °C.
Chemical Aging of EPDM [38]	Chemical (NaOH, H_3_PO_4_, NaClO)	NaOH, NaClO, H_3_PO_4_, 65 °C	Accelerated crosslinking in NaOH and H_3_PO_4_ exposure, reduction in glass transition temperature, oxidation damage. Increased crosslink density in NaClO exposure during compression.
Multi-Stress Aging of EPDM and Silicone Rubber [39]	Electrical + Mechanical	Electrical stress: 11.5 kV/mm; Mechanical stretching: 0%, 35%, 65%; Aging times: 0, 50, 100 h	For EPDM, surface damage occurred but internal properties remained stable. Mechanical properties declined by less than 20%, and the crosslinking degree remained stable.
Chemical Degradation of SR, EPDM, FKM in PEMFC Environment [40]	Chemical + Thermal	80 °C, Sulfuric Acid (pH = 3–4), Nafion® Accelerated Solution	SR experienced degradation with surface cracking and filler loss; FKM showed the best stability; EPDM showed stable mechanical properties.
Chemical and Thermal Degradation of PEFC Sealants—FKM, EPDM, Silicone Rubber [41]	Chemical + Thermal	60–80 °C, Sulfuric Acid (pH = 3.35)	EPDM showed good chemical stability. Silicone exhibited degradation in the form of weight loss. FKM showed the highest thermal stability.

#### 2.3.1. Hyperparameter Tuning in Ensemble Models

Optimizing ensemble models requires careful hyperparameter tuning, which can significantly affect model accuracy and efficiency. Two popular tuning methods are as follows:

- GridSearchCV: an exhaustive search over predefined hyperparameter grids, selecting the best combination based on a performance metric, though computationally intensive for large search spaces.
- RandomSearchCV: samples hyperparameters from a defined distribution, finding near-optimal solutions efficiently by avoiding exhaustive searches; ideal for large datasets.

For ensemble models like RF and Gradient Boosting, hyperparameters such as the number of estimators, maximum depth of trees, learning rate, and regularization parameters are commonly tuned using these methods. Properly selecting hyperparameters can significantly impact ensemble models’ accuracy and computational efficiency.

#### 2.3.2. Related Works in Applications of Different Machine Learning Models in Material Science

EL has demonstrated success in material science, enhancing predictive accuracy in fault detection, aging classification, and stress distribution. This study employs stacking to combine models such as SVM and RF, optimized through RandomSearchCV and GridSearchCV. As PEMFCs continue to emerge as sustainable energy solutions, it is paramount to ensure operational reliability through advanced diagnostic methods. This section reviews recent advancements, emphasizing machine learning, EL, and predictive methodologies for PEMFC fault detection and degradation analysis. Timely fault diagnosis is crucial for maintaining PEMFC reliability. Wang et al. developed a fault diagnosis model by combining segmented cell technology with a dual-input convolutional neural network (CNN), achieving over 98.5% accuracy in identifying flooding and drying faults. This approach leverages current distribution and sensor data, highlighting segmented cell technology’s potential for enhanced water management in PEMFCs [42]. While fault diagnosis is vital, prescriptive maintenance frameworks further extend PEMFC efficiency and durability. Gibey et al. developed a prescriptive maintenance model that utilizes RF for real-time diagnostics and BiLSTM and BiESN for predicting Remaining Useful Life (RUL) based on voltage degradation. Operating within a hybrid Cloud–Edge architecture, this framework enables in situ, online PEMFC maintenance [43]. The accurate performance prediction of PEMFCs, mainly the polarization curve, is essential for optimizing zero-emission electro-hydrogen generators. Soufian et al. proposed an AI-based model, integrating kernel principal component analysis and mutual information for feature selection, followed by XGBRegressor with Bayesian optimization. Tested on industrial PEMFC data, this model outperformed conventional methods, setting a benchmark for AI-driven predictive maintenance in fuel cells [44]. Addressing durability and corrosion challenges in PEMFCs, Madhavan et al. used machine learning, specifically extreme gradient boosting (XGB) and artificial neural networks (ANN), to predict the corrosion resistance of diamond-like carbon (DLC)-coated metallic bipolar plates (MBPs). ANN achieved $R^{2}>0.98$ for both corrosion current density and impedance parameters, underscoring the value of ML for rapid MBP performance prediction [45]. Due to complex physical interactions in PEMFC systems, Zhang et al. developed an ensemble fault diagnosis method, integrating five distinct algorithms. This method achieves over 95% precision, recall, and F-measure across various PEMFC faults. This ensemble method enhances diagnostic stability, especially in cases of sensor failure, across diverse operational environments [46]. Shin et al. applied models like DNN, RF, and SVM across 18 specific PEMFC faults in thermal and air management. The DNN model excelled with an F1-score of 0.987 for fault detection and 0.942 for fault diagnosis. This study highlights the robustness of ML-based fault diagnosis and its integration potential for PEMFC monitoring [47]. The accurate degradation prediction of PEMFCs is essential for health management. Yu et al. introduced the UTE-MLSTM model, a framework incorporating Time-Varying Filtered Empirical Mode Decomposition (TVF-EMD), Uniform Manifold Approximation and Projection (UMAP), and Mogrifier LSTM (M-LSTM). This model significantly improved predictive accuracy, offering a stable solution for PEMFC health monitoring [48]. Ozdemir et al. demonstrated the efficacy of ML models like SVM, MLP, and RF for Proton Exchange Membrane Water Electrolyzer (PEMWE) systems in hydrogen production. Their model predicted vital parameters such as hydrogen flow rate and current density. SVM achieved a Mean Absolute Error (MAE) of 0.0317 for current density and 0.0671 for hydrogen flow rate, underscoring ML’s role in optimizing PEMWE performance [49]. Effective fault detection in Polymer Electrolyte Fuel Cells (PEFCs) also supports system health and operational efficiency. Melo et al. employed ML and DL models across seven classifiers to diagnose PEFC faults in a dataset of 182,156 records. Models like logistic regression (LR), KNN, DT, RF, and NB demonstrated high accuracy with lower computational costs, indicating ML’s promise for PEFC maintenance and design optimization [50]. Collectively, these studies illustrate the rapid advancements in machine learning and deep learning applications for PEMFC and PEMWE systems, spanning fault diagnosis, predictive maintenance, and performance optimization. From precise diagnostic frameworks and prescriptive maintenance models to AI-driven performance predictions, these approaches underscore the immense potential of data-driven techniques to address the complex challenges in hydrogen energy systems. Integrating diverse ML and DL methodologies enhances fault detection and predictive accuracy and opens avenues for more resilient, efficient, and sustainable fuel-cell technologies. Building on these advancements, the present study aims to further refine diagnostic and predictive models, contributing to the evolution of robust maintenance frameworks for PEMFC and PEMWE applications.

## 3. Hyperelastic Modeling Approach

**Figure 1:**
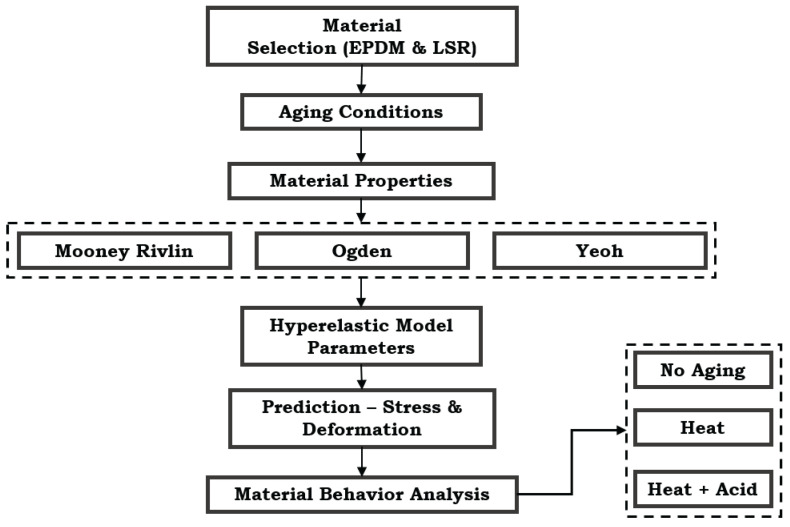
Proposed hyperelastic modeling approach for PEMFC gasket materials.

This section details the methodological framework to investigate the mechanical performance and predictive maintenance of EPDM and LSR gasket materials in PEMFC systems. As depicted in Figure 1, the proposed methodology involves a multi-step framework integrating hyperelastic modeling, simulation, and EL to comprehensively assess material degradation under varied aging conditions. The framework employs Mooney–Rivlin, Yeoh, and Ogden models to capture the stress–strain characteristics of EPDM and LSR, followed by a machine learning approach to predict material behavior. This combined approach facilitates predictive maintenance by enabling the accurate classification of aging effects on gasket materials and supports optimized model selection for performance prediction in PEMFC applications.

### 3.1. Materials and Aging Conditions

This study evaluates EPDM and LSR, elastomers commonly used in PEMFC gaskets. Known for their durability under challenging conditions, EPDM resists heat and weathering, while LSR withstands harsh chemicals. The aging conditions applied in this study, including sustained heat at 95 °C, are designed to simulate high-temperature operational stresses typical of PEMFC applications, excluding low-temperature or cold-start scenarios. Their mechanical performance under three aging conditions is analyzed using hyperelastic models to capture the stress–strain behavior:

- No Aging: baseline condition with no aging exposure.
- Heat-Aging: 95 °C for 3000 h, simulating high-temperature operation.
- Heat- + Sulfuric-Acid-Aging: 95 °C with 5% sulfuric acid exposure ($H_{2}{\mathrm{SO}}_{4}$) for 3000 h, representing severe thermal and chemical degradation.

### 3.2. Hyperelastic Models

To characterize stress–strain behavior, three hyperelastic models were chosen for their suitability in predicting elastomeric response under large deformations:

1. Mooney–Rivlin Model: suitable for moderate strains, this model’s strain energy function depends on the first two invariants of the deformation tensor.
2. Yeoh Model: using only the first invariant performs well for materials under large deformations.
3. Ogden Model: employing principal stretches, this effectively captures non-linear elastic behavior at large deformations.

### 3.3. Stress and Deformation Metrics

To assess the mechanical performance of the materials under different aging conditions, the following metrics are analyzed:

- von Mises Stress: this metric evaluates the equivalent stress distribution in the material, providing insights into areas of potential failure under load.
- Contact Stress: this is evaluated at the interface between the gasket and the mating components to understand the material’s ability to maintain sealing integrity.
- Height (Deformation): the deformation of the gasket under load is measured to assess how much compression or elongation the material undergoes during operation. This is critical for ensuring proper sealing in PEMFC applications.

### 3.4. Simulation Setup and Tools

**Figure 2:**
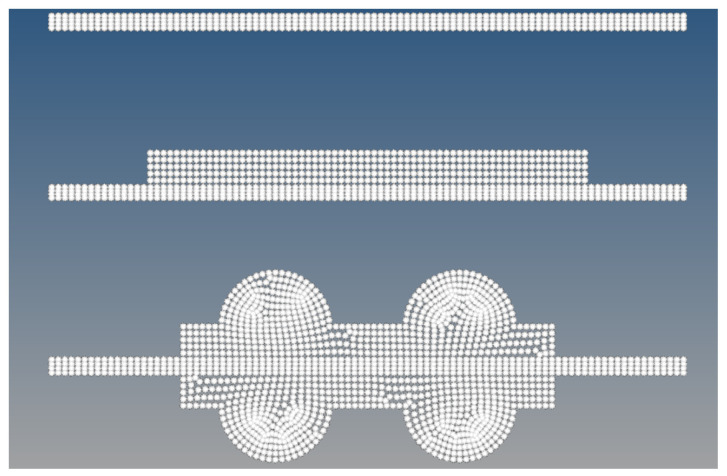
Mesh generation using Hypermesh for the gasket material simulation.

The simulations were conducted using MSC MARC 2011, Version 2011.1, MSC Software Corporation, Newport Beach, CA, USA, a robust finite element analysis (FEA) software suite particularly suited for non-linear materials and hyperelastic modeling. The computations were performed on a desktop system with an AMD Ryzen 9 3950X 16-core processor with a base speed of 3.5 GHz and 32 GB of RAM. This configuration provided ample computational power to handle the intensive simulation tasks required for hyperelastic material analysis. Each analysis took approximately 30 min, allowing for the efficient processing of multiple simulation runs. The gasket has a cross-sectional profile with a width of 4.8 mm and a height of 1.85 mm, composed of multiple layers: a 0.09 mm Pi film layer, a 0.34 mm anode layer, and a 1.42 mm cathode layer. This layered structure is essential for capturing the complex interactions and mechanical responses under varying aging conditions. Figure 2 illustrates the structured mesh generated for the gasket geometry, which was imported into MARC for further analysis to ensure accuracy in capturing the mechanical behavior of EPDM and LSR materials under these conditions. The following steps detail the setup of the simulation environment:

- Importing preprocessed files: The initial geometry and mesh were generated using HYPERMESH, and the files were subsequently imported into MSC MARC for further analysis. ABAQUS file formats (.inp) were utilized to ensure compatibility and smooth integration between the preprocessing tools and the MARC solver. This preprocessing stage ensures a high-quality mesh and accurate geometry representation for analyzing EPDM and LSR gaskets.
- Element type selection: Our precision and expertise were demonstrated in selecting specific element types to model the mechanical behavior of gasket materials and other components in the simulation. For planar dimensions, we used element ID types (Type 80 for gaskets and Type 11 for contact elements), which are well suited for the simulation of large deformations typical in hyperelastic materials such as EPDM and LSR. The material properties of steel components were also defined, using the typical values for Young’s modulus (210 GPa) and Poisson’s ratio (0.3) for an accurate interaction between rigid and deformable bodies [51].
- Contact interaction setup: In the simulation, we defined deformable contact bodies between critical components, including the interaction between the gasket and mating surfaces. We utilized MARC’s advanced contact modeling capabilities to ensure accurate force transfer and deformation behavior between interacting surfaces, highlighting the advanced tools and techniques employed in the simulation.
- Solver and post-processing: The non-linear solver in MSC MARC was employed to handle large deformations and non-linear material behavior. After solving the finite element model, post-processing tools extracted vital performance metrics, including von Mises stress, contact stress, and deformation for each aging condition. The results were then visualized and compared to experimental data for validation.

### 3.5. Hyperelastic Model Parameters and Simulation Computing Environment

The FEA modeling for EPDM and LSR gasket materials is based on the selected hyperelastic models: Mooney–Rivlin, Yeoh, and Ogden. Each model’s parameters were optimized to accurately reflect the materials’ mechanical behavior under various aging conditions. The FEA modeling results, shown in Table 2, outline the fitted parameters for each hyperelastic model obtained at a reference temperature of 95 °C. These parameters are inputs in the subsequent simulations and are crucial for understanding the materials’ stress–strain responses in predictive maintenance applications. These parameters are critical for describing the stress–strain behavior of the materials in the simulations. The Mooney–Rivlin model utilizes the constants $C_{10}$, $C_{01}$, and $C_{11}$, while the Yeoh model incorporates $C_{10}$, $C_{20}$, and $C_{30}$. The Ogden model includes moduli and exponents for three terms, capturing the complex material behavior under large deformations.

**Figure 3:**
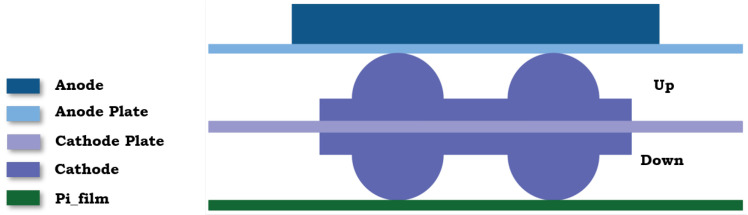
A cross-sectional profile of the gasket assembly, showing the Pi film layer, cathode, and anode sections, and supporting plates for structural stability.

The gasket assembly consists of multiple layers that provide structural integrity and effective sealing under operational conditions. As shown in Figure 3, the assembly includes a Pi film layer at the base, followed by a structured cathode section with separate cathode-up and cathode-down layers, a central cathode plate, and finally, an anode plate supporting the anode layer. This configuration ensures optimal mechanical performance and compatibility with the aging conditions under study.

## 4. EL Model Approach

**Figure 4:**
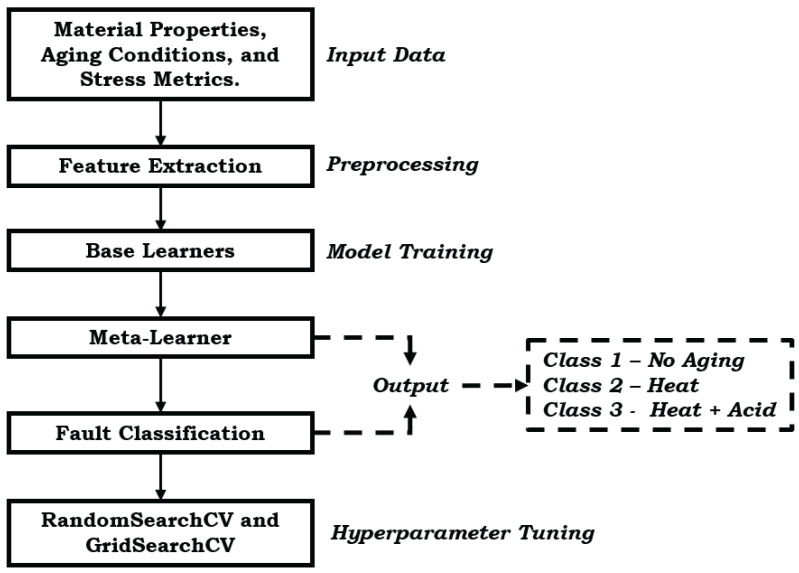
The framework of the stacking ensemble model.

This study utilizes a stacked EL model, combining Random Forest (RF) and Support Vector Machine (SVM) classifiers to improve classification accuracy for aging effects in EPDM and LSR gasket materials. The architecture, illustrated in Figure 4, integrates RF and SVM base learners with a meta-classifier, aiming to capture complex patterns in the data and improve robustness against data variability. EL combines multiple models, or base learners, to achieve greater predictive accuracy and robustness than individual models. By leveraging the complementary strengths of different algorithms, EL reduces the risk of overfitting, making it well suited for classifying material behavior under aging conditions in EPDM and LSR gaskets. This supports predictive maintenance by accurately modeling stress distribution and aging effects, contributing to the enhanced durability of PEMFC gasket materials. Specifically, this stacking ensemble model uses SVM and RF to improve accuracy in classifying stress distributions and aging effects. It enables proactive maintenance interventions by accurately predicting material degradation and reducing false positives and negatives in fault detection. The stacking framework integrates predictions from SVM and RF through an additional RF classifier as the meta-learner. This setup maximizes classification accuracy for non-linear, high-dimensional data:

- SVM: is chosen for its ability to handle high-dimensional spaces and robustness in finding optimal decision boundaries.
- RF: included for its ensemble approach, which reduces variance by constructing multiple decision trees trained on different data subsets.

The meta-learner, an RF classifier, combines the base learners’ predictions, optimally weighting their outputs to enhance overall prediction accuracy.

The parameters for the SVM and RF classifiers used in the stacked EL are detailed in Table 3. These parameters were optimized through hyperparameter tuning using RandomSearchCV and GridSearchCV, allowing adjustments to kernel types, regularization for SVM, and the number of estimators and maximum depth for RF. The tuning process involved cross-validation to enhance robustness, with RandomSearchCV initially exploring a broader parameter range, followed by GridSearchCV to fine-tune the optimal configurations. The complete machine learning pipeline, including data preprocessing, model training, and evaluation, is outlined in the pseudocode provided in the Appendix A.

Common metrics, including accuracy, precision, recall, and F1-score, were used to evaluate the model’s performance. The mathematical expressions for these metrics are as follows:

(5) $$\mathrm{Accuracy}\;=\;\frac{\mathrm{TP}+\mathrm{TN}}{\mathrm{TP}+\mathrm{TN}+\mathrm{FP}+\mathrm{FN}}$$

(6) $$\mathrm{Precision}\;=\;\frac{\mathrm{TP}}{\mathrm{TP}+\mathrm{FP}}$$

(7) $$\mathrm{Recall}\;=\;\frac{\mathrm{TP}}{\mathrm{TP}+\mathrm{FN}}$$

(8) $$F1\;\mathrm{Score}\;=\;2\cdot \frac{\mathrm{Precision}\times \mathrm{Recall}}{\mathrm{Precision}+\mathrm{Recall}}$$

where TP = true positives, TN = true negatives, FP = false positives, and FN = false negatives. To evaluate the effectiveness of the stacked EL model, classification performance was measured using standard metrics, with particular emphasis on achieving high accuracy and balanced precision and recall. A model F1-score above 0.80 was set as the benchmark for reliable performance, ensuring the accurate classification of degradation patterns while minimizing false positives and false negatives. This classification standard supports the EL model’s role in predictive maintenance, providing reliable assessments of PEMFC gasket conditions under aging effects in EPDM and LSR materials [52].

### EL Computing Environment

The machine learning experiments were conducted on a desktop system with the following specifications: AMD Ryzen 5 5600 6-Core Processor, with a base speed of 3.5 GHz, six cores, 12 logical processors, and a cache configuration that significantly enhanced its performance: 384 KB L1 cache, 3.0 MB L2 cache, and 32.0 MB L3 cache. The system also had 56 GB of RAM, running on Windows 10. The study utilized Python 3.8, Scikit-Learn 0.24, and TensorFlow 2.4. Depending on model complexity, hyperparameter tuning with RandomSearchCV and GridSearchCV took approximately 3–4 h per model.

## 5. Results and Discussion

### 5.1. Contact Stress

**Figure 5:**
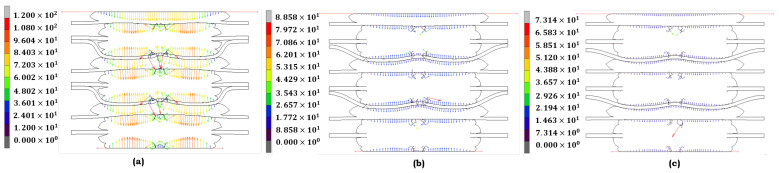
Contact-stress contour distribution of **EPDM** under different aging conditions: (**a**) no aging (Ogden); (**b**) heat (Mooney–Rivlin); and (**c**) heat + sulfuric acid (Yeoh).

Each model is selected for the EPDM material under different aging conditions based on how effectively it represents its behavior under stress. If the material has not undergone any aging (Figure 5a), the Ogden model is the most appropriate choice. The contact stress distribution is relatively even, with moderate peaks, indicating that the material retains much of its original elasticity. The Ogden model captures this behavior, reflecting EPDM’s stability without aging factors like heat or acid. When EPDM is subjected to heat (Figure 5b), the Mooney–Rivlin model comes into play. It effectively portrays the material’s loss of elasticity as the contact stress becomes more concentrated, especially in the central region. The model’s selection for this condition is significant, as it accurately reflects how heat affects the material, resulting in a moderate increase in stress. This portrayal of the material’s behavior under heat is a key aspect of the model’s effectiveness. Regarding combined heat and acid exposure (Figure 5c), EPDM shows significant peaks in contact stress, indicating substantial structural breakdown. The stress distribution is no longer uniform, with localized peaks pointing to severe material fatigue. The Yeoh model is the best fit here, designed to handle rubber-like materials under large strains. The Yeoh model accurately reflects EPDM’s highly non-linear response when subjected to heat and acid, showing the material’s progressive failure under extreme conditions.

**Figure 6:**
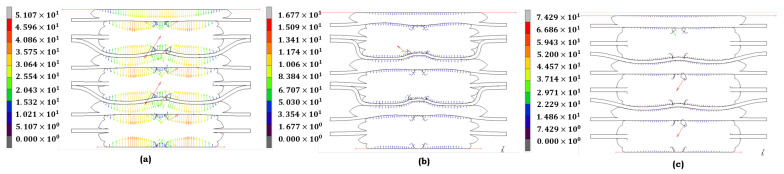
Contact-stress contour distribution of **LSR** under different aging conditions: (**a**) no aging (Mooney–Rivlin); (**b**) heat (Ogden); and (**c**) heat + sulfuric acid (Yeoh).

The Mooney–Rivlin model accurately represents its behavior when the LSR material is not aged (Figure 6a). LSR exhibits a relatively uniform stress distribution, indicating its high elasticity and resilience. The Mooney–Rivlin model captures this stable state, making it the best fit for LSR in its unaged condition, where the material’s elastic properties are fully intact. Stress concentrations appear when LSR is exposed to heat (Figure 6b). The Ogden model is chosen here because it best represents the slight loss of elasticity due to thermal exposure. Ogden’s capability to manage moderate deformations aligns with the observed behavior, where the material shows localized peaks but continues to perform within acceptable limits. The Yeoh model again proves its worth in the most severe condition, where LSR is exposed to heat and acid (Figure 6c). It effectively captures the severe structural degradation LSR experiences under these conditions, where the material’s elastic limits are exceeded. The model’s ability to simulate large strains and significant material responses is crucial in this context, as it accurately portrays the substantial deformation and sharp peaks in stress that suggest critical fatigue. The model selection for both EPDM and LSR is grounded in their respective stress responses to different aging conditions. For unaged materials, the Ogden model accurately captures the balanced stress distributions. Under moderate heat exposure, the Mooney–Rivlin model portrays the slight degradation of elasticity. Finally, the Yeoh model is the most suitable for conditions involving combined heat and acid exposure, as it effectively captures the non-linear behavior and material fatigue that both EPDM and LSR exhibit in extreme environments. Each figure complements the discussion by illustrating the distinct stress distributions for the models chosen under each condition.

### 5.2. Von Mises Stress

**Figure 7:**
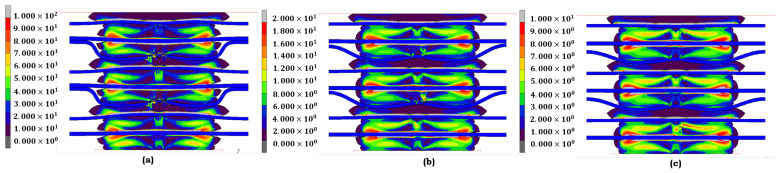
von Mises stress distribution of **EPDM** under different aging conditions: (**a**) no aging (Ogden); (**b**) heat (Mooney–Rivlin); and (**c**) heat + sulfuric acid (Yeoh).

Each model is meticulously chosen for the EPDM material based on its precise representation of its structural response to stress across various aging conditions. This rigorous selection process ensures the accuracy and reliability of our findings. The Ogden model (Figure 7a) accurately depicts stress distribution under no aging conditions. EPDM retains much elasticity, and the stress remains evenly distributed. The Ogden model reflects this behavior, capturing EPDM’s stable structural integrity under these conditions, where no significant degradation has occurred. The Mooney–Rivlin model (Figure 7b) best fits when heat is applied. Under heat exposure, EPDM experiences an increase in stress concentration, particularly in the center regions, indicating some loss of elasticity. The Mooney–Rivlin model effectively portrays this moderate degradation, illustrating how heat impacts the material’s ability to maintain its original mechanical properties. Under combined heat and acid exposure, the Yeoh model (Figure 7c) is the most appropriate choice. EPDM exhibits highly localized stress peaks, indicating significant structural breakdown. The Yeoh model captures the extreme, non-linear material response under this condition, reflecting the progressive failure as EPDM loses its ability to handle strain effectively.

**Figure 8:**
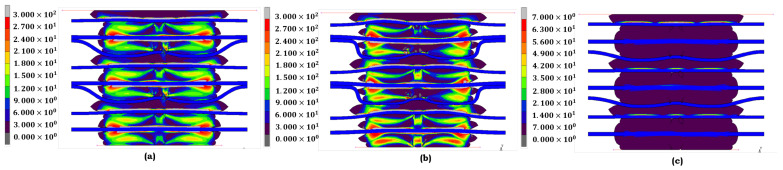
von Mises stress distribution of **LSR** under different aging conditions: (**a**) no aging (Mooney–Rivlin), (**b**) heat (Ogden); and (**c**) heat + sulfuric acid (Yeoh).

The selection for the LSR material is similarly grounded in the material’s response to von Mises stress under different conditions. When LSR is not aged, the Mooney–Rivlin model (Figure 8a) best represents its stress behavior. LSR exhibits a relatively uniform stress distribution, indicating that the material’s elasticity is intact. The Mooney–Rivlin model accurately captures this stable state, making it the best fit for LSR in its unaged form. The Ogden model (Figure 8b) is the most suitable under heat exposure. LSR shows the onset of stress concentration, especially in specific localized regions. The Ogden model’s ability to handle moderate deformation makes it well suited to depict LSR’s performance, where heat causes a slight degradation in elasticity. Finally, when LSR is exposed to heat and acid, the Yeoh model (Figure 8c) provides the most precise representation of stress behavior. The material undergoes significant deformation, and the Yeoh model’s capacity to capture large strains accurately represents the severe fatigue and degradation of LSR under extreme conditions.

The models selected for both EPDM and LSR under von Mises stress conditions demonstrate their adaptability to different aging conditions. The Ogden and Mooney–Rivlin models offer the best fit for unaged materials due to their capability to represent balanced and stable stress distributions. Under moderate heat exposure, the Mooney–Rivlin and Ogden models accurately portray the elasticity loss. Finally, the Yeoh model consistently captures the severe degradation and material fatigue for both EPDM and LSR for the combined heat and acid condition. Each model complements the specific aging condition, with the figures provided illustrating the stress distribution that guided the model selection.

### 5.3. Comparison of EPDM and LSR for Each Aging Condition Using Different Models

The stress and deformation behaviors of EPDM and LSR under different aging conditions, as evaluated using the Mooney–Rivlin, Ogden, and Yeoh models, reveal significant findings. These distinct differences between the two materials, reflecting their respective resistances to stress and deformation, are of utmost importance in materials science and polymer engineering. The comparison of von Mises stress, contact stress, and deformation (height) for EPDM and LSR under various aging conditions, using the Mooney–Rivlin, Ogden, and Yeoh models, reveals critical insights into the material behavior of both elastomers. As shown in Table 4, the von Mises stress values for EPDM are generally lower than those for LSR under all conditions. For example, under no aging, the von Mises stress at the 1st cathode-up location for EPDM is 14.96 MPa using the Mooney–Rivlin model, while LSR shows a significantly higher value of 24.17 MPa under the same conditions. The same trend holds for the heat and heat + sulfuric acid conditions. This indicates that LSR tends to experience more significant stress than EPDM, likely due to its more elastic nature. The Mooney–Rivlin model consistently shows the highest stress for both materials, with the Ogden model yielding the next highest values, followed by the Yeoh model. Table 5 provides contact stress comparisons, further emphasizing the higher stress endured by LSR compared to EPDM. For instance, at the 1st cathode-down location under heat conditions, LSR shows a contact stress of 296.93 MPa with the Ogden model, whereas EPDM records a much lower value of 22.53 MPa. Across all models and aging conditions, LSR shows a higher contact stress response, which aligns with its greater flexibility and susceptibility to deformation under stress. The Ogden model yields the highest contact stress across the board, followed by the Mooney–Rivlin and Yeoh models. Deformation measurements presented in Table 6 show that, despite the higher stress experienced by LSR, its deformation remains similar to that of EPDM. Under heat + sulfuric acid conditions, the 1st cathode-up position shows a deformation of 0.3851 mm for EPDM (Mooney–Rivlin model) and 0.3800 mm for LSR. This indicates that, while LSR tends to deform more under stress than EPDM, the overall deformation across different models and conditions remains relatively consistent for both materials. The Ogden model generally records the highest deformation values, reflecting its capacity to capture more significant elastic deformations.

The results show that LSR tends to experience more significant stress and contact stress than EPDM across all conditions and models, though both materials exhibit similar deformation values. These findings suggest that LSR is more responsive to stress and suitable for flexible applications. At the same time, EPDM maintains its integrity under high-stress conditions, making it ideal for applications prioritizing rigidity and durability.

The Mooney–Rivlin, Ogden, and Yeoh models each provide distinct insights into the mechanical behavior of LSR and EPDM materials under different aging conditions. They present unique and overlapping observations on von Mises stress, contact stress, and material height.

**Figure A1:**
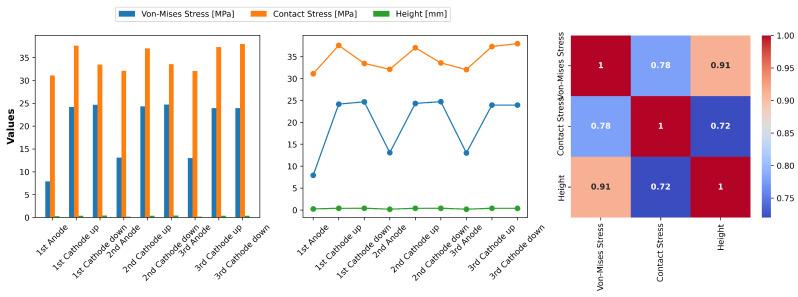
Mooney–Rivlin model analysis of von Mises stress, contact stress, and height (deformation) for LSR materials under no aging conditions.

**Figure A2:**
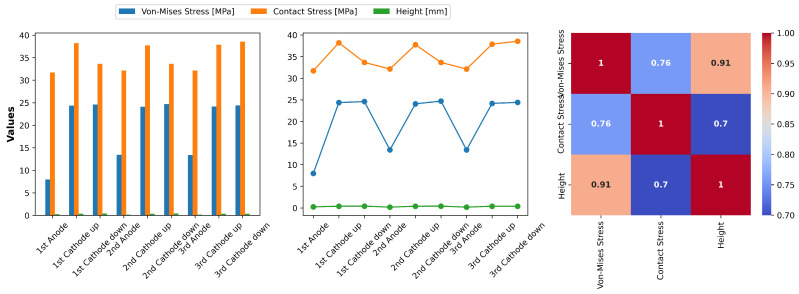
Mooney–Rivlin model analysis of von Mises stress, contact stress, and height (deformation) for LSR materials under heat-aging conditions.

**Figure A3:**
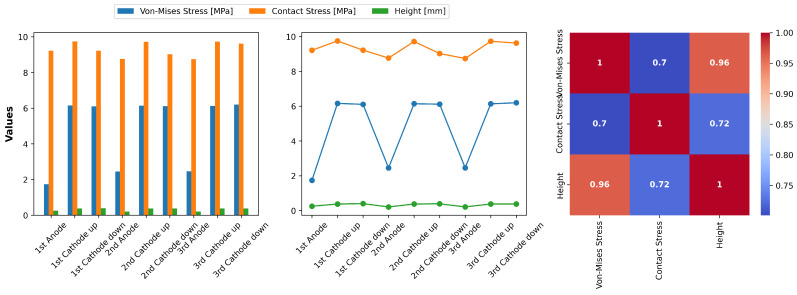
Mooney–Rivlin model analysis of von Mises stress, contact stress, and height (deformation) for LSR materials under heat- + sulfuric-acid-aging conditions.

Starting with LSR, the Mooney–Rivlin model offers a foundational understanding of stress distribution under its “No Aging” conditions (Figure A1). Here, we observe baseline stress levels across all tested locations, with notably higher contact stress in the 1st cathode positions, hinting at potential stress concentration zones in unaged LSR. This scenario shifts under “Heat Aging” (Figure A2), where von Mises stress significantly increases, especially at the cathode positions, suggesting that thermal exposure induces a degree of rigidity in LSR. However, under the “Heat + Sulfuric Acid Aging” conditions (Figure A3), there is a slight reduction in both von Mises and contact stress, indicating that sulfuric acid may counteract the thermal rigidity, softening the material and thereby reducing its stress resistance.

**Figure A4:**
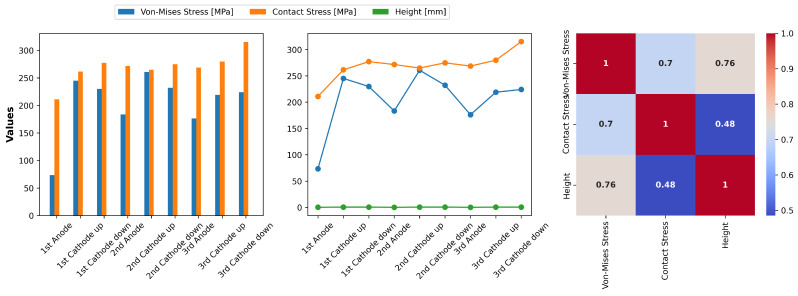
Ogden model analysis of von Mises stress, contact stress, and height (deformation) for LSR materials under no aging conditions.

**Figure A5:**
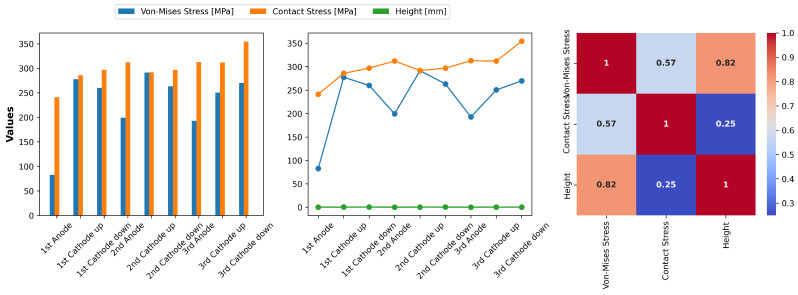
Ogden model analysis of von Mises stress, contact stress, and height (deformation) for LSR materials under heat-aging conditions.

**Figure A6:**
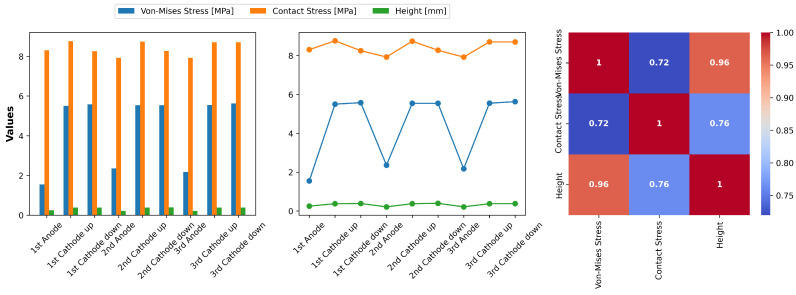
Ogden model analysis of von Mises stress, contact stress, and height (deformation) for LSR materials under heat- + sulfuric-acid-aging conditions.

The Ogden model, in the “No Aging” state (Figure A4), presents a similar baseline to Mooney–Rivlin but shows heightened sensitivity to elasticity changes under “Heat Aging” (Figure A5). Both von Mises and contact stress increase markedly at the anode and cathode locations, implying that heat exacerbates material rigidity when elasticity-sensitive parameters are considered. Upon exposure to “Heat + Sulfuric Acid Aging” (Figure A6), stress levels drop substantially compared to the heat-only condition, reinforcing the notion that sulfuric acid induces a degradation in LSR’s structural integrity, counteracting the rigidifying effect of heat. This degradation aligns with the Mooney–Rivlin findings, bolstering the hypothesis that sulfuric acid compromises the material’s resilience.

**Figure A7:**
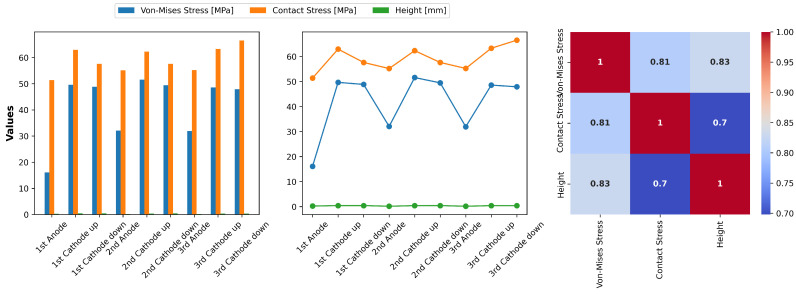
Yeoh model analysis of von Mises stress, contact stress, and height (deformation) for LSR materials under no aging conditions.

**Figure A8:**
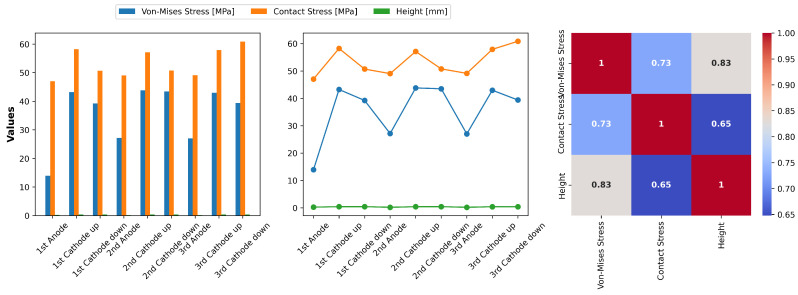
Yeoh model analysis of von Mises stress, contact stress, and height (deformation) for LSR materials under heat-aging conditions.

**Figure A9:**
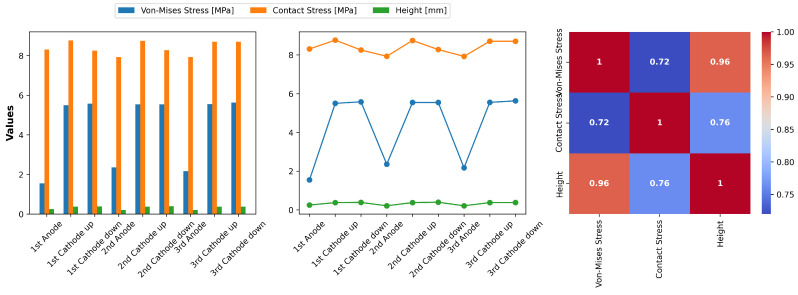
Yeoh model analysis of von Mises stress, contact stress, and height (deformation) for LSR materials under heat- + sulfuric-acid-aging conditions.

The Yeoh model complements this analysis with further insights into LSR’s deformation tendencies. Under the “No Aging” conditions (Figure A7), the distribution of height and stress levels mirrors that of previous models. Under “Heat Aging” (Figure A8), both stress types rise, confirming that thermal exposure stiffens the material. However, “Heat + Sulfuric Acid Aging” (Figure A9) reveals reduced stress values and subtle deformations in height, implying that acid exposure not only reduces rigidity but also affects the material’s deformation capacity, likely due to chemical softening. This pattern suggests that the Yeoh model captures not only stress variations but also subtle shifts in material deformation, aligning well with observed chemical degradation effects.

**Figure A10:**
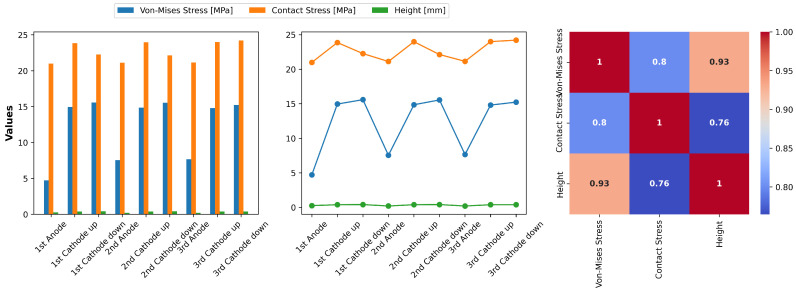
Mooney–Rivlin model analysis of von Mises stress, contact stress, and height (deformation) for EPDM material under no aging conditions.

**Figure A11:**
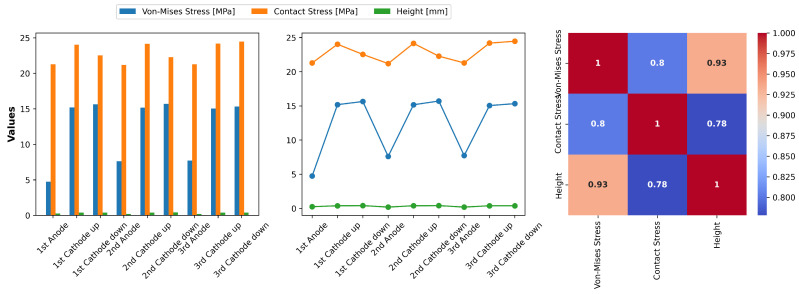
Mooney–Rivlin model analysis of von Mises stress, contact stress, and height (deformation) for EPDM material under heat-aging conditions.

**Figure A12:**
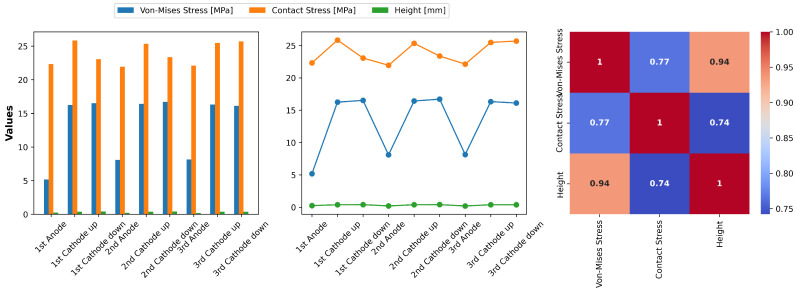
Mooney–Rivlin model analysis of von Mises stress, contact stress, and height (deformation) for EPDM material under heat- + sulfuric-acid-aging conditions.

Turning to EPDM, the Mooney–Rivlin model (Figure A10, Figure A11 and Figure A12) consistently reflects von Mises stress responsiveness to aging, showing EPDM’s higher stress capacity in no aging compared to heat- and acid-aged conditions. The strong correlation between von Mises and contact stress underscores an even distribution of external pressure within the material. However, the model displays limited sensitivity to changes in height, holding these values nearly constant, which suggests it may be less effective at detecting physical deformations over time.

**Figure A13:**
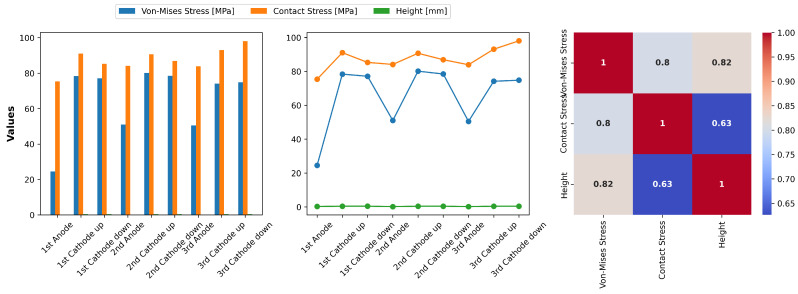
Ogden model analysis of von Mises stress, contact stress, and height (deformation) for EPDM material under no aging conditions.

**Figure A14:**
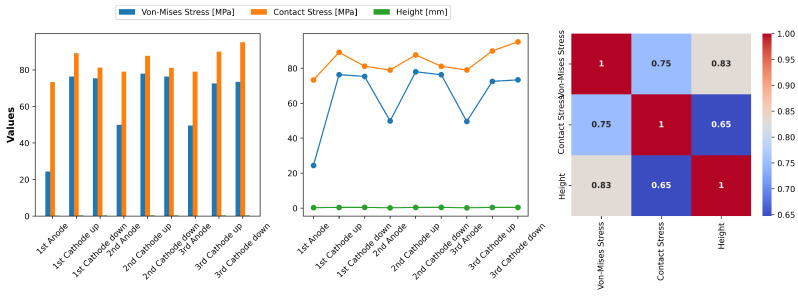
Ogden model analysis of von Mises stress, contact stress, and height (deformation) for EPDM material under heat-aging conditions.

**Figure A15:**
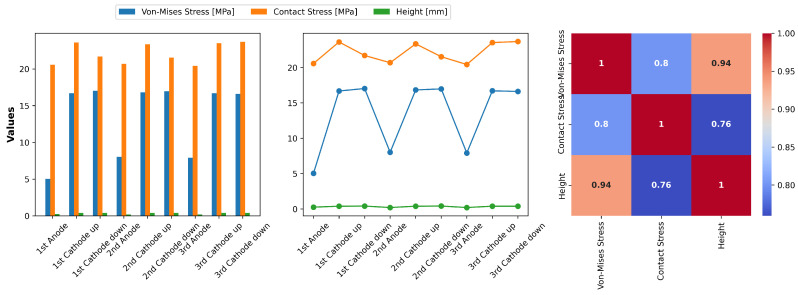
Ogden model analysis of von Mises stress, contact stress, and height (deformation) for EPDM material under heat- + sulfuric-acid-aging conditions.

The Ogden model (Figure A13, Figure A14 and Figure A15) offers a sharper focus on height changes, particularly under prolonged heat- and sulfuric-acid-aging. This model’s pronounced contact stress response underscores increased EPDM’s rigidity under combined aging conditions, indicating that it might be more apt for failure prediction under extreme conditions. The Ogden model’s tendency to emphasize stress concentration more than the Mooney–Rivlin model implies its utility in scenarios where chemical exposure affects the physical properties of EPDM.

**Figure A16:**
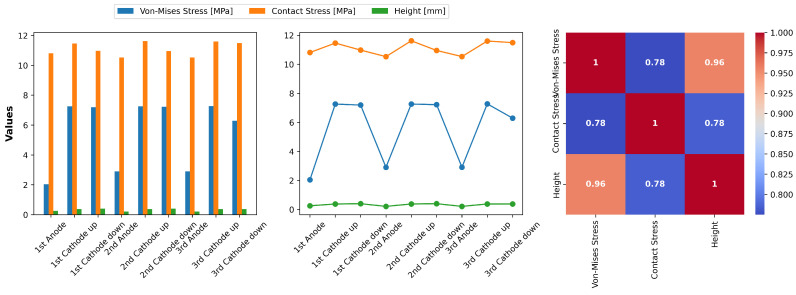
Yeoh model analysis of von Mises stress, contact stress, and height (deformation) for EPDM material under no aging conditions.

**Figure A17:**
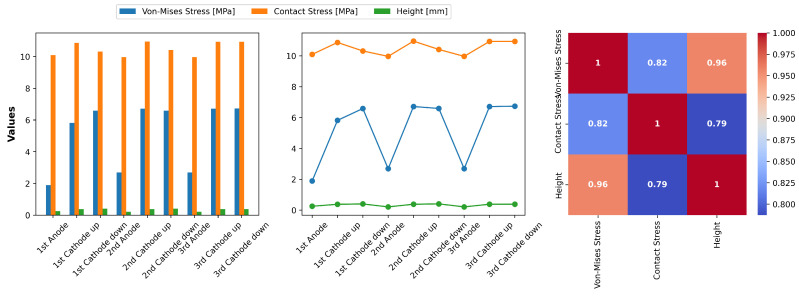
Yeoh model analysis of von Mises stress, contact stress, and height (deformation) for EPDM material under heat-aging conditions.

**Figure A18:**
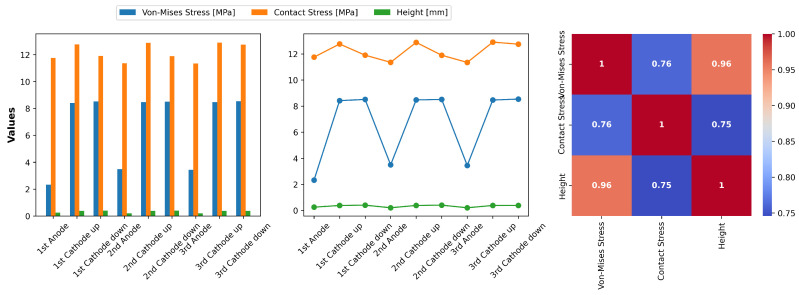
Yeoh model analysis of von Mises stress, contact stress, and height (deformation) for EPDM material under heat- + sulfuric-acid-aging conditions.

In contrast, the Yeoh model (Figure A16, Figure A17 and Figure A18) demonstrates a balanced sensitivity to stress and height changes across all aging conditions. Its ability to capture stress variations comparable to the Ogden model while interpreting subtle height shifts highlights its versatility and reliability. The Yeoh model’s stress resilience under severe aging conditions, particularly with acid exposure, suggests its efficacy in long-term durability predictions for EPDM, making it particularly useful when both mechanical and chemical degradation factors are of interest.

The LSR and EPDM analyses across the Mooney–Rivlin, Ogden, and Yeoh models illustrate a cohesive narrative of how heat- and sulfuric-acid-aging differentially affect these materials. These findings have significant implications for engineering applications, providing a comprehensive understanding of how these materials perform under varying environmental stressors. The Mooney–Rivlin model offers a foundational overview of stress behaviors; the Ogden model emphasizes elasticity and failure points under severe conditions; and the Yeoh model balances stress and deformation sensitivity. For EPDM, the Mooney–Rivlin model offers a simplified perspective with limited deformation insights, the Ogden model is suited for extreme aging sensitivity, and the Yeoh model’s balanced response aids in understanding long-term durability. These combined insights underscore the importance of a multi-model approach in understanding LSR and EPDM performance and reliability.

### 5.4. EL Performance Metrics

The EL model’s performance was evaluated using standard classification metrics, including accuracy, precision, recall, and F1-score. These metrics were computed for the model and classifications of aging types for EPDM and LSR materials.

The LSR model-type and aging-type classification results demonstrate strong and reliable performance across both search methods. For model type, RandomSearchCV achieved an accuracy of 0.95, with the optimal hyperparameters tuned for SVM using a linear kernel. GridSearchCV produced a similar result with a slight reduction in accuracy at 0.94. These scores indicate satisfactory balance across precision, recall, and F1-score across all classes (Table 7). For aging type, RandomSearchCV outperformed slightly, delivering a higher test accuracy of 0.98, particularly effective in identifying class 1 with high precision and recall values. GridSearchCV, with an accuracy of 0.97, maintained consistent performance across classes, showing that both methods effectively capture the aging effect on LSR materials (Table 8).

In the case of EPDM, the classification results for both model and aging types also show robustness. For model type, both RandomSearchCV and GridSearchCV achieved a test accuracy of 0.84, confirming the model’s effectiveness in recognizing EPDM material types (Table 9). When focused on the aging type, RandomSearchCV achieved a slightly higher accuracy of 0.81, with a high recall of 0.93 in class 0, highlighting the model’s ability to detect specific aging conditions accurately. GridSearchCV’s accuracy was slightly lower, at 0.80, but showed consistent results, confirming its effectiveness (Table 10).

These results, highlighted in the tables, underscore the effectiveness of both search methods in delivering high classification accuracy, precision, and recall. RandomSearchCV displayed a marginal edge in optimizing complex aging classifications, demonstrating its slight advantage in capturing nuanced patterns in aging-related tasks across EPDM and LSR materials.

**Figure 9:**
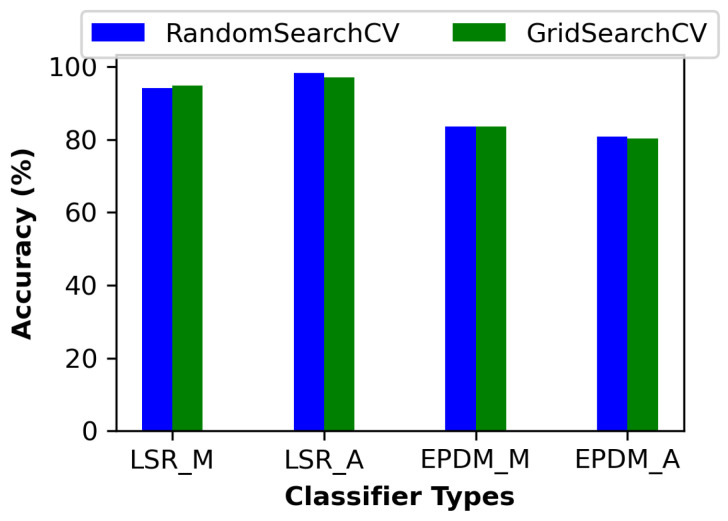
Comparison of classification accuracy for LSR and EPDM materials using RandomSearchCV and GridSearchCV for model type (M) and aging type (A) classification.

The comparison of classifier performance on LSR and EPDM datasets, as shown in Table 11 and illustrated in Figure 9, highlights the clear advantage of using a stacked ensemble model combining RF and SVM, particularly for accurately classifying both model type and aging type. The alternative classifiers used for comparison against the stacked ensemble model were randomly selected from the literature to represent a diverse range of commonly applied machine learning techniques, ensuring a robust benchmarking context and further underscoring the superior performance of the ensemble approach in this study.

The stacked model achieves significantly higher accuracy than individual classifiers like XGBoost, MLP, and DNN. For LSR, the stacked model attains 95% accuracy for the model type and 98% for the aging type, underscoring its strong capability to capture complex data patterns. In contrast, DNN, which performs moderately well for LSR aging-type classification at 70% accuracy, falls short compared to the ensemble approach. XGBoost and MLP achieve lower accuracies of approximately 70% and 62% for model type and aging type, respectively, demonstrating their limited effectiveness with LSR data.

Similarly, in the EPDM dataset, the stacked model maintains its superior performance, achieving 84% accuracy for the model type and 83% for the aging type. While DNN shows promise in aging-type classification with 76% accuracy, it remains less consistent in precision and recall than the stacked model. XGBoost reaches 69% accuracy for the EPDM model type but continues to underperform relative to the ensemble model. The bar chart further illustrates the impact of hyperparameter search techniques, with RandomSearchCV slightly outperforming GridSearchCV in most cases. Notably, RandomSearchCV helps the stacked model reach its highest scores, such as 98% accuracy for the LSR aging type and 83% for the EPDM aging type, underscoring its utility in fine-tuning ensemble models for complex, non-linear classification tasks, such as distinguishing aging conditions in different material types.

In summary, the stacked ensemble approach, integrating the predictive strengths of RF and SVM, is the most effective classifier for both datasets and classification tasks. Its impressively high accuracy and reliability make it a valuable tool for predictive maintenance applications in PEMFC gasket materials, providing a robust solution for anticipating material degradation. This analysis emphasizes that while individual models like DNN may be viable alternatives in specific cases, ensemble methods deliver superior performance, ensuring robustness and precision in classification tasks crucial for industrial applications.

### 5.5. Comparison with the Recent Literature on Stress and Aging Performance

The findings from this study align closely with recent advancements in PEMFC gasket research, reassuringly validating the role of optimized material selection and structural resilience under various stress and aging conditions. In examining contact stress, it was observed that LSR consistently maintained higher values compared to EPDM across all aging scenarios, particularly under the conditions of heat and sulfuric acid exposure. This robustness suggests that LSR is well suited for reliable sealing applications in high-stress environments. This result corresponds with the findings in [13], who reported that half-circular gasket geometries enhanced airtightness and durability, especially under extremely low temperatures. While Yoo’s work focused on geometry, the high contact-stress retention observed in LSR suggests that material resilience can further complement structural design choices to ensure PEMFC integrity under fluctuating conditions. Regarding von Mises stress, the study demonstrated that LSR sustains higher internal stresses than EPDM, indicating that it can withstand greater mechanical loads without yielding. This result aligns with the rate-dependent aging model developed in [16], which revealed that EPDM’s stress tolerance diminishes over prolonged aging, particularly under thermal and mechanical stress. The results of this study affirm that LSR’s higher von Mises stress retention makes it a more resilient choice for PEMFC applications that involve high-load demands over time. The aging effects in this study revealed that both EPDM and LSR degrade under prolonged exposure to heat and sulfuric acid; however, LSR showed more excellent stability by maintaining higher stress values and experiencing less deformation. This aligns with findings in [17], who reported an increased compression set and reduced mechanical resilience in EPDM under thermal cycling, emphasizing LSR’s superior performance under temperature fluctuations. Additionally, the study in [15] highlighted system-level PEMFC design improvements that enhance durability through optimized mass transfer and thermal management. This strategy could be further supported by integrating durable gasket materials like LSR to extend the PEMFC’s lifespan. These findings demonstrate that LSR’s superior contact and von Mises stress retention under aging conditions make it more suitable than EPDM for PEMFC applications that require reliable sealing performance and resilience under fluctuating stress. This consistency with the recent literature validates the potential of LSR as a practical choice in PEMFC gasket applications. More importantly, it contributes to predictive maintenance strategies and informed material selection, offering hope for enhanced fuel cell performance and longevity.

## 6. Conclusions, Limitations and Future Directions

This study comprehensively examined the mechanical behavior of EPDM and LSR gasket materials under various aging conditions using hyperelastic modeling and machine learning, providing insights crucial for the broader fields of materials science, engineering, and industrial maintenance. The differential stress and deformation responses observed for EPDM and LSR materials, particularly under heat and sulfuric acid exposure, underscore the nuanced requirements of gasket materials in high-stress applications. Our results demonstrate that EPDM exhibits superior stability, making it particularly suitable for high-stress environments where long-term durability is essential. Conversely, LSR, while highly flexible, shows greater susceptibility to stress concentrations and deformation, potentially limiting its application in harsh environments. These insights are valuable for improving gasket selection for PEMFC systems and supporting proactive maintenance strategies that enhance fuel-cell reliability and longevity in industrial applications. By focusing on gasket material performance at high operational temperatures, this study provides practical insights for PEMFC applications in demanding environments. It contributes to material selection and maintenance strategies that support PEMFC durability under prolonged thermal stress, empowering professionals to make informed decisions in their field. Integrating machine learning, specifically ensemble learning techniques such as stacking with Random Forest and SVM, further enhances predictive accuracy regarding material behavior under different aging conditions. Hyperparameter optimization with RandomSearchCV and GridSearchCV enabled the precise classification of EPDM and LSR materials, positioning the ensemble approach as a critical tool for predicting material degradation and informing maintenance strategies in real-world settings. While the research yields promising results, it is essential to recognize its limitations. While effective in capturing specific material behaviors under targeted aging conditions, the hyperelastic models may only partially account for the multi-stress factors present in real-world scenarios. These factors include UV exposure, prolonged mechanical wear, and other environmental stressors. Furthermore, although ensemble learning demonstrated high accuracy, its computational complexity may pose challenges for real-time predictive maintenance, highlighting the need for future model efficiency improvements. Another limitation pertains to the dataset, which focuses on a limited set of aging conditions. Expanding the dataset to include a broader range of environmental exposures and material compositions could yield more universally applicable insights. This potential for future research, along with the incorporation of digital twin technology to simulate real-time material performance, further enhancing predictive accuracy, should inspire professionals in the field.

## Figures and Tables

**Table 2:** FEA material model parameters under aging conditions.

Model	Parameters	Value	
EPDM	Mooney–Rivlin	$C_{10}$	1.89472 × $10^{-9}$
		$C_{01}$	0.609227
		$C_{11}$	0.194325
	Yeoh	$C_{10}$	0.559296
		$C_{20}$	0.026558
		$C_{30}$	0.00294048
	Ogden	Modulus 1	−0.664004
		Modulus 2	−2.20126 × $10^{-5}$
		Modulus 3	0.0757264
		Exponent 1	−3.93474
		Exponent 2	−0.0664976
		Exponent 3	4.91726
LSR	Mooney–Rivlin	$C_{10}$	0.430276
		$C_{01}$	0.0162594
		$C_{11}$	0.016835
	Yeoh	$C_{10}$	0.475585
		$C_{20}$	0.00318065
		$C_{30}$	2.74909 × $10^{-13}$
	Ogden	Modulus 1	−0.402385
		Modulus 2	0.626279
		Modulus 3	7.91621 × $10^{-13}$
		Exponent 1	−0.686773
		Exponent 2	2.41526
		Exponent 3	15.2995

**Table 3:** Stacked ensemble model parameters for SVM and RF.

ML Classifier	Major Functional Parameters	Parameter Values
SVM	Regularization (C)	0.1, 1, 10
	Kernel	Linear, RBF
	Gamma (γ)	Auto
RF	Number of Estimators (*n* estimators)	50, 100, 200
	Max Depth	10, 20, None
	Random State	42

**Table 4:** Comparison of von Mises stress (MPa) for EPDM and LSR across different aging conditions using Mooney–Rivlin, Ogden, and Yeoh models.

Location	EPDM			LSR		
	Mooney–Rivlin	Ogden	Yeoh	Mooney–Rivlin	Ogden	Yeoh
No Aging						
1st Anode	4.71	24.54	2.04	7.93	73.46	16.11
1st Cathode Up	14.96	78.40	7.26	24.17	245.18	49.64
1st Cathode Down	15.59	77.12	7.19	24.68	229.84	48.83
Heat						
1st Anode	4.74	24.41	1.89	7.98	82.61	13.92
1st Cathode Up	15.18	76.30	5.82	24.37	277.51	43.22
1st Cathode Down	15.64	75.30	6.58	24.61	260.11	39.23
Heat + Sulfuric Acid						
1st Anode	5.19	5.04	2.33	1.74	1.55	1.50
1st Cathode Up	16.25	16.68	8.42	6.16	5.50	5.41
1st Cathode Down	16.52	17.03	8.51	6.10	5.58	5.34

**Table 5:** Comparison of contact stress (MPa) for EPDM and LSR across different aging conditions using Mooney–Rivlin, Ogden, and Yeoh models.

Location	EPDM			LSR		
	Mooney–Rivlin	Ogden	Yeoh	Mooney–Rivlin	Ogden	Yeoh
No Aging						
1st Anode	20.99	75.38	10.81	31.11	211.01	51.35
1st Cathode Up	23.86	91.07	11.46	37.57	261.49	62.93
1st Cathode Down	22.26	85.31	10.98	33.45	277.17	57.61
Heat						
1st Anode	21.29	73.32	10.09	31.72	241.29	47.03
1st Cathode Up	24.02	89.09	10.86	38.20	285.88	58.21
1st Cathode Down	22.53	81.14	10.31	33.66	296.93	50.69
Heat + Sulfuric Acid						
1st Anode	22.32	20.57	11.75	9.21	8.31	8.10
1st Cathode Up	25.83	23.61	12.76	9.75	8.76	8.50
1st Cathode Down	23.07	21.70	11.90	9.22	8.25	8.18

**Table 6:** Comparison of height (deformation) (mm) for EPDM and LSR across different aging conditions using Mooney–Rivlin, Ogden, and Yeoh models.

Location	EPDM			LSR		
	Mooney–Rivlin	Ogden	Yeoh	Mooney–Rivlin	Ogden	Yeoh
No Aging						
1st Anode	0.2522	0.2472	0.2520	0.2518	0.1987	0.2494
1st Cathode Up	0.3848	0.4103	0.3755	0.3908	0.4140	0.4021
1st Cathode Down	0.4046	0.4139	0.3949	0.4061	0.4220	0.4097
Heat						
1st Anode	0.2521	0.2442	0.2524	0.2517	0.2028	0.2520
1st Cathode Up	0.3841	0.4055	0.3800	0.3905	0.4175	0.4025
1st Cathode Down	0.4043	0.4146	0.4013	0.4108	0.4254	0.4202
Heat + Sulfuric Acid						
1st Anode	0.2508	0.2515	0.2510	0.2493	0.2497	0.2505
1st Cathode Up	0.3851	0.3838	0.3802	0.3746	0.3754	0.3782
1st Cathode Down	0.4054	0.4028	0.4029	0.3990	0.3844	0.3966

**Table 7:** Classification results for LSR model type using RandomSearchCV and GridSearchCV.

Search Method	Class	Precision	Recall	F1-Score
RandomSearchCV	0	0.94	0.94	0.99
	1	0.98	0.91	0.94
	2	0.93	0.97	0.90
Test Accuracy				0.95
Best Accuracy				0.88
Best Hyperparameters		{’svm__kernel’: ’linear’, ’svm__C’: 1, ’rf__n_estimators’: 200, ’rf__max_depth’: None, ’final_estimator__n_estimators’: 100, ’final_estimator__max_depth’: 10}		
GridSearchCV	0	0.91	0.90	0.95
	1	0.98	0.98	0.93
	2	0.93	0.93	0.93
Test Accuracy				0.94
Best Accuracy				0.90
Best Hyperparameters		{’final_estimator__max_depth’: 10, ’final_estimator__n_estimators’: 200, ’rf__max_depth’: 20, ’rf__n_estimators’: 200, ’svm__C’: 1, ’svm__kernel’: ’linear’}		

**Table 8:** Classification results for LSR aging type using RandomSearchCV and GridSearchCV.

Search Method	Class	Precision	Recall	F1-Score
RandomSearchCV	0	0.90	0.98	0.94
	1	0.92	0.92	0.92
	2	0.91	0.95	0.98
Test Accuracy				0.98
Best Accuracy				0.93
Best Hyperparameters		{’svm__kernel’: ’rbf’, ’svm__C’: 0.1, ’rf__n_estimators’: 200, ’rf__max_depth’: 20, ’final_estimator__n_estimators’: 50, ’final_estimator__max_depth’: 20}		
GridSearchCV	0	0.81	0.85	0.83
	1	0.91	0.88	0.90
	2	0.86	0.87	0.86
Test Accuracy				0.97
Best Accuracy				0.91
Best Hyperparameters		{’final_estimator__max_depth’: 10, ’final_estimator__n_estimators’: 200, ’rf__max_depth’: None, ’rf__n_estimators’: 50, ’svm__C’: 0.1, ’svm__kernel’: ’rbf’}		

**Table 9:** Classification results for EPDM model type using RandomSearchCV and GridSearchCV.

Search Method	Class	Precision	Recall	F1-Score
RandomSearchCV	0	0.80	0.83	0.86
	1	0.85	0.82	0.85
	2	0.82	0.87	0.89
Test Accuracy				0.84
Best Accuracy				0.83
Best Hyperparameters		{’svm__kernel’: ’rbf’, ’svm__C’: 10, ’rf__n_estimators’: 200, ’rf__max_depth’: None, ’final_estimator__n_estimators’: 100, ’final_estimator__max_depth’: None}		
GridSearchCV	0	0.80	0.83	0.86
	1	0.85	0.82	0.85
	2	0.82	0.87	0.89
Test Accuracy				0.84
Best Accuracy				0.83
Best Hyperparameters		{’final_estimator__max_depth’: None, ’final_estimator__n_estimators’: 100, ’rf__max_depth’: None, ’rf__n_estimators’: 200, ’svm__C’: 10, ’svm__kernel’: ’rbf’}		

**Table 10:** Classification results for EPDM aging type using RandomSearchCV and GridSearchCV.

Search Method	Class	Precision	Recall	F1-Score
RandomSearchCV	0	0.88	0.93	0.81
	1	0.95	0.89	0.85
	2	0.81	0.81	0.85
Test Accuracy				0.81
Best Accuracy				0.81
Best Hyperparameters		{’svm__kernel’: ’rbf’, ’svm__C’: 10, ’rf__n_estimators’: 200, ’rf__max_depth’: None, ’final_estimator__n_estimators’: 100, ’final_estimator__max_depth’: None}		
GridSearchCV	0	0.85	0.90	0.89
	1	0.90	0.89	0.84
	2	0.83	0.81	0.87
Test Accuracy				0.80
Best Accuracy				0.83
Best Hyperparameters		{’final_estimator__max_depth’: 10, ’final_estimator__n_estimators’: 200, ’rf__max_depth’: 20, ’rf__n_estimators’: 200, ’svm__C’: 10, ’svm__kernel’: ’rbf’}		

**Table 11:** Comparison of model performance on LSR and EPDM datasets for model-type and aging-type classification.

Model	Target	Dataset	Accuracy	Precision	Recall	F1-Score
XGBoost	Model Type	LSR	0.70	0.71	0.70	0.70
	Model Type	EPDM	0.69	0.67	0.69	0.68
	Aging Type	LSR	0.62	0.62	0.62	0.61
	Aging Type	EPDM	0.62	0.67	0.68	0.67
MLP	Model Type	LSR	0.59	0.66	0.59	0.59
	Model Type	EPDM	0.55	0.60	0.55	0.53
	Aging Type	LSR	0.67	0.60	0.67	0.65
	Aging Type	EPDM	0.58	0.61	0.62	0.64
DNN	Model Type	LSR	0.62	0.65	0.62	0.61
	Model Type	EPDM	0.69	0.66	0.69	0.66
	Aging Type	LSR	0.70	0.77	0.70	0.71
	Aging Type	EPDM	0.76	0.78	0.76	0.76
Stacked Model	Model Type	LSR	0.95	0.93	0.97	0.90
	Model Type	EPDM	0.84	0.82	0.87	0.89
	Aging Type	LSR	0.98	0.91	0.95	0.98
	Aging Type	EPDM	0.83	0.81	0.83	0.87

## Data Availability

The data presented in this study are available on request from the corresponding author. The data are not publicly available due to laboratory regulations.

## Appendix A

**Algorithm A1:** Machine learning pipeline for model-type and aging-type classification
**Input**: LSR dataset $D_{\mathrm{LSR}}\;=\;\left\{X_{\mathrm{LSR}},y_{\mathrm{model}\_\mathrm{LSR}},y_{\mathrm{aging}\_\mathrm{LSR}}\right\}$, EPDM dataset $D_{\mathrm{EPDM}}\;=\;\left\{X_{\mathrm{EPDM}},y_{\mathrm{model}\_\mathrm{EPDM}},y_{\mathrm{aging}\_\mathrm{EPDM}}\right\}$ **Output**: Final accuracy $A_{\mathrm{final}}$, classification report, and confusion matrix $C_{\mathrm{final}}$ **_1_** Load datasets $D_{\mathrm{LSR}}$ and $D_{\mathrm{EPDM}}$ from CSV files; **_2_** Concatenate features: $X_{\mathrm{model}}\leftarrow \left[X_{\mathrm{LSR}},X_{\mathrm{EPDM}}\right]$; **_3_** Concatenate model-type labels: $y_{\mathrm{model}}\leftarrow \left[y_{\mathrm{model}\_\mathrm{LSR}},y_{\mathrm{model}\_\mathrm{EPDM}}\right]$; **_4_** Encode model-type labels: $y_{\mathrm{model}\_\mathrm{encoded}}\leftarrow \mathrm{LE}\left(y_{\mathrm{model}}\right)$; **_5_** Normalize features: $X_{\mathrm{model}\_\mathrm{scaled}}\leftarrow \mathrm{SC}\left(X_{\mathrm{model}}\right)$; **_6_** Concatenate features: $X_{\mathrm{aging}}\leftarrow \left[X_{\mathrm{LSR}},X_{\mathrm{EPDM}}\right]$; **_7_** Concatenate aging-type labels: $y_{\mathrm{aging}}\leftarrow \left[y_{\mathrm{aging}\_\mathrm{LSR}},y_{\mathrm{aging}\_\mathrm{EPDM}}\right]$; **_8_** Encode aging-type labels: $y_{\mathrm{aging}\_\mathrm{encoded}}\leftarrow \mathrm{LE}\left(y_{\mathrm{aging}}\right)$; **_9_** Normalize features: $X_{\mathrm{aging}\_\mathrm{scaled}}\leftarrow \mathrm{SC}\left(X_{\mathrm{aging}}\right)$; **_10_** Split $X_{\mathrm{model}\_\mathrm{scaled}},y_{\mathrm{model}\_\mathrm{encoded}}$ and $X_{\mathrm{aging}\_\mathrm{scaled}},y_{\mathrm{aging}\_\mathrm{encoded}}$ into 80–20 training and testing sets; **Input**: Model *M*, training and testing sets for model-type or aging-type classification task **Output**: Performance metrics: accuracy *A*, classification report, and confusion matrix *C* **_11_** Train model *M* on training data $X_{\mathrm{train}},y_{\mathrm{train}}$ to minimize the loss function *L*; **_12_** $$M\leftarrow \arg\min L(M\left(X_{\mathrm{train}}\right),y_{\mathrm{train}})$$ Predict test labels ${\hat{y}}_{\mathrm{test}}$ on $X_{\mathrm{test}}$:; **_13_** $${\hat{y}}_{\mathrm{test}}\;=\;M\left(X_{\mathrm{test}}\right)$$ Calculate accuracy *A*:; **_14_** $$A\;=\;\frac{1}{n}\sum_{i=1}^{n}⊮\left({\hat{y}}_{i}\;=\;y_{i}\right)$$ Output classification report and confusion matrix *C*; **_15_** Define and initialize models for both classification tasks as follows: - **XGBoost**: Define with default hyperparameters. - **Multilayer Perceptron (MLP)**: Define with 100 hidden units and ReLU activation. - **Deep Neural Network (DNN)**: Define with hidden layers [150,100,50]. - **Stacked Model (RF + SVM)**: Use Random Forest (RF) and Support Vector Machine (SVM) as base estimators, with an RF as the final estimator. **Input**: Stacked model $M_{\mathrm{stacked}}$, Parameter grid *G* **Output**: Optimal parameters $G^{*}$ and best model $M_{\mathrm{best}}$ **_16_** Perform a randomized search on $M_{\mathrm{stacked}}$ with grid *G*; **_17_** **for** *each parameter configuration g∈G* **do**( **_18_** ˪ Evaluate $M_{\mathrm{stacked}}\left(g\right)$ on the training set and compute accuracy A(g); **_19_** Set $G^{*}\leftarrow \arg\max A\left(g\right)$ and update best model $M_{\mathrm{best}}\;=\;M_{\mathrm{stacked}}\left(G^{*}\right)$; **Input**: Optimized stacked model $M_{\mathrm{best}}$, testing set $X_{\mathrm{test}},y_{\mathrm{test}}$ **Output**: Final accuracy $A_{\mathrm{final}}$, classification report, and confusion matrix $C_{\mathrm{final}}$ **_20_** Use $M_{\mathrm{best}}$ to predict labels ${\hat{y}}_{\mathrm{test}}$ on test data:; **_21_** $${\hat{y}}_{\mathrm{test}}\;=\;M_{\mathrm{best}}\left(X_{\mathrm{test}}\right)$$ Calculate and output final accuracy $A_{\mathrm{final}}$, classification report, and confusion matrix $C_{\mathrm{final}}$; **_22_** **return** $A_{\mathrm{final}},C_{\mathrm{final}}$
